# Minimally Invasive Excision of an Oral Lipoma of the Upper Lip Using a 980nm Diode Laser: A Case Report

**DOI:** 10.7759/cureus.78722

**Published:** 2025-02-08

**Authors:** Salvatore L La Terra, Gianluigi Caccianiga, Faisal Alzahrani, Faris M. Alabeedi, Francesco Buoncristiani

**Affiliations:** 1 Regenerative Cellular Therapy, Faculty of Natural Health Science, Selinus University of Science and Literature, London, GBR; 2 Periodontology, Oral Surgery, Oral Medicine, "La Terra" Private Dental Clinic, Ragusa, ITA; 3 Translational Medicine, University of Ferrara, Ferrara, ITA; 4 Oral and Maxillofacial Surgery, Royal Armed Forces Medical Service, Riyadh, SAU; 5 Oral Surgery, College of Medicine and Dentistry, Ulster University, Birmingham, GBR; 6 Maxillofacial Surgery and Diagnostic Science, Faculty of Dentistry, Najran University, Najran, SAU; 7 Oral Surgery, "Buoncristiani" Private Dental Clinic, Livorno, ITA

**Keywords:** benign lesion, diode laser surgery, excision, lipoma, upper lip

## Abstract

Lipomas of the upper lip are among the rarest adipose tissue neoplasms in the oral cavity. The standard treatment for lipomas involves surgical removal, which can be performed using conventional or laser techniques. This case report discusses a 71-year-old male patient with swelling on the left side of his upper lip, clinically diagnosed as an oral lipoma. The lesion was surgically removed under local anesthesia using a combination of diode laser surgery and a blunt scalpel. This approach facilitated the complete removal of the lesion with reduced operating time, minimal damage to surrounding tissues, and uneventful postoperative healing. Diode laser surgery for benign lipomas presents an effective alternative to traditional methods. The precision and minimal tissue damage it offers are particularly beneficial in aesthetic areas, such as the lips.

## Introduction

The advent of laser technology has significantly transformed various medical and dental practices, offering innovative solutions for diverse clinical challenges. In dentistry, lasers are indispensable tools for a broad spectrum of procedures, including low-level laser therapy, laser bandaging, photodynamic therapy, and soft-tissue excision [1]. Among the lasers used for soft-tissue procedures, diode lasers stand out due to their affinity for melanin and hemoglobin, enabling precise cutting combined with simultaneous tissue coagulation. This dual action minimizes bleeding, enhances surgical visibility, reduces the need for manual hemostasis, and improves overall patient comfort [2].

The minimally invasive nature of diode lasers contributes to reduced tissue trauma, faster healing, and diminished postoperative discomfort and swelling [3]. Additionally, precise laser energy delivery often results in smaller incisions and better cosmetic outcomes, which are particularly advantageous in aesthetically sensitive areas, such as the upper lip [1].

Lipomas are relatively common benign neoplasms of the head and neck region but are rare in the upper lip, constituting only 2% of all oral lesions [4]. These slow-growing, non-invasive tumors are highly differentiated, often presenting as painless, sessile, or pedunculated masses with pale-colored mucosa [5]. While the buccal mucosa [6] and gingiva [7] are the most frequent sites of occurrence, upper lip lipomas are less commonly reported. The etiology of lipomas remains uncertain, with factors such as mechanical irritation, chronic inflammation, obesity, and chromosomal abnormalities implicated in their development [2].

Traditional treatment modalities for oral lipomas include excision using scalpel blades, cryosurgery, and electrocautery. However, these methods are associated with several disadvantages, such as excessive bleeding, risk of infection, scar formation, postoperative pain, and delayed wound healing [2, 4, 8]. To overcome these limitations, diode lasers have been introduced as a modern alternative since their application in dentistry in 1999. The diode laser, a solid-state semiconductor device with wavelengths ranging from 810 nm to 980 nm, is widely recognized for its superior handling, pain control, minimal postoperative complications, reduced surgical time, and cost-effectiveness [5, 7].

This case report highlights the successful application of a diode laser for the excision of an oral lipoma in the upper lip, emphasizing its efficacy, precision, and potential advantages over conventional surgical techniques.

## Case presentation

Patient information

A 71-year-old male patient was referred to the office for a consultation and visit with the chief complaint of “difficulty in chewing and speaking, and aesthetic issue because of the presence of a large-sized mass at the level of the upper lip.” The painless mass, located on the left side of the upper lip, had persisted for six months. The patient was a non-smoker, non-alcoholic, and in good general health, with no history of surgery, recurrent mouth ulcers, fever, or systemic diseases.

Clinical evaluation

**Figure 1:**
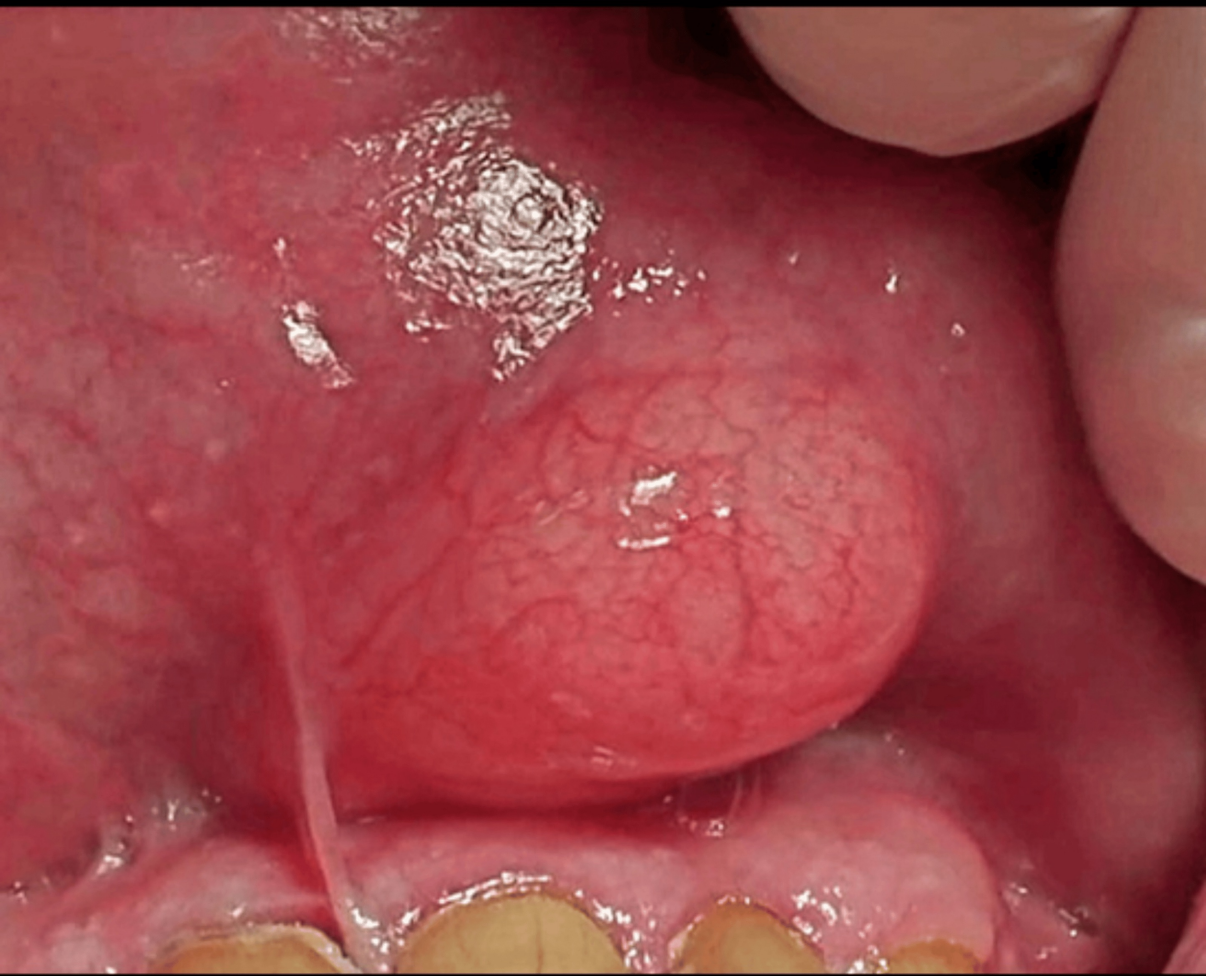
A well-circumscribed, sessile, smooth-surfaced intra-oral mass.

Extraoral examination revealed asymmetry of the left side of the upper lip due to significant swelling. Intraoral examination showed an asymptomatic, painless, slow-growing, well-circumscribed submucosal lesion on the left side of the upper lip. The lesion, sessile in nature, was covered by normal mucosa, had a tense-elastic consistency, and appeared yellowish, which the patient found bothersome. The mass measured approximately 2 mm x 1.3 mm in diameter (Figure 1). No evidence of dental trauma or overhanging restorations was observed.

Based on clinical examination, the patient was provisionally diagnosed with a lipoma. To confirm the diagnosis and exclude high-flow vascular lesions, echocolor Doppler imaging and ultrasonography were performed. Ultrasonography revealed a hypoechoic, gelatinous mass measuring 1.9 x 1.3 cm, consistent with a benign lesion. Echocolor Doppler analysis excluded the presence of high-flow vascular lesions. Treatment options, including surgical excision using diode laser surgery, were discussed with the patient, and informed consent was obtained.

Surgical approach

**Figure 2:**
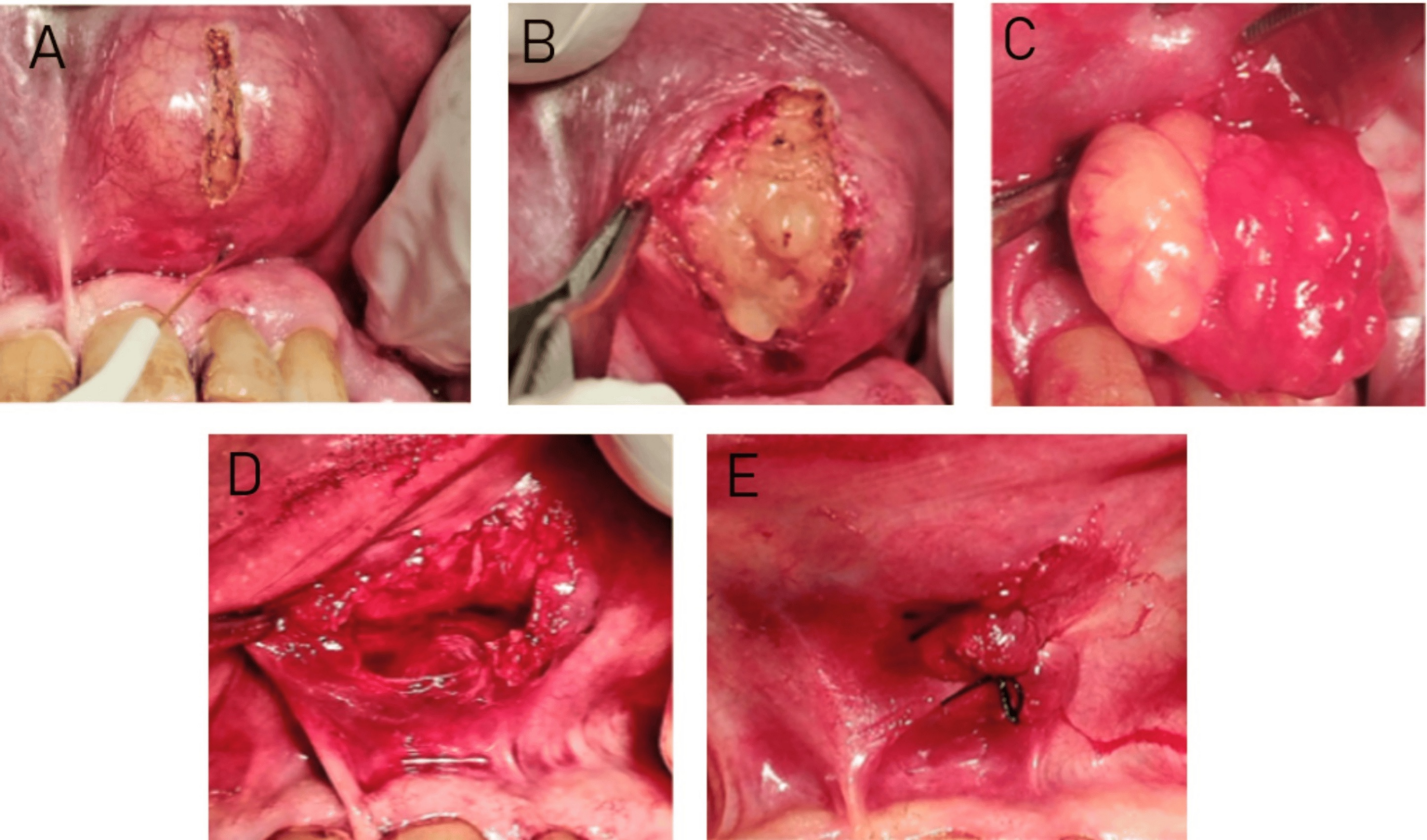
Surgical excision of oral lipoma using a 980 nm diode laser. A) Blunt dissection with the laser. B) Lesion separation with a blunt instrument. C) Digital pressure applied for lesion removal. D) Excised lesion post-laser surgery. E) Interrupted sutures placed post-surgery.

Prior to the procedure, mouth antisepsis was performed using a 0.2% chlorhexidine solution. With a clinical diagnosis of oral lipoma, the lesion was surgically excised under local anesthesia (3% mepivacaine). A diode laser (Litemedics Prime, Milan, Italy) with a 25 kHz frequency, 12W peak power, and a 320 µm fiber tip operating at a wavelength of 980 nm and a 2W output power in continuous wave contact mode was used for the surgical enucleation. The laser produced a small vertical incision over the top of the lesion, minimizing tissue carbonization (Figure 2A). During the procedure, the surgical site was kept dry, and surgical gauze was used to stabilize the lip and limit movement.

**Figure 3:**
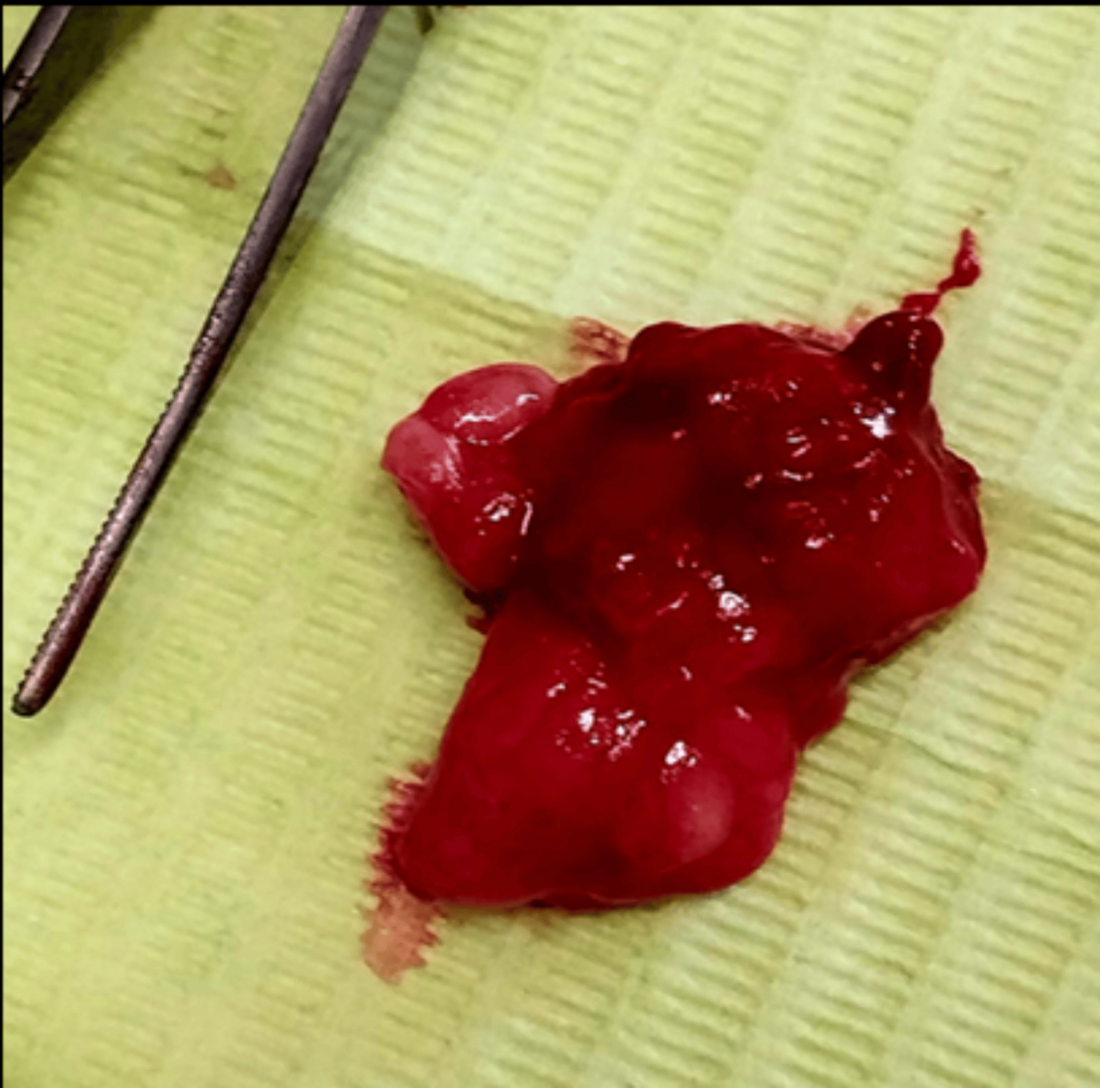
Complete excised mass of oral lipoma measuring 2 x1.3 mm.

Surgical separation of tissue layers was achieved through blunt dissection and gentle traction using blunt instruments, facilitating submucosal exposure of the lesion (Figure 2B). Digital pressure further aided in the enucleation of the lesion (squeeze technique) (Figure 2C). Minimal to no bleeding was observed at the surgical site (Figure 2D). Because of the size of the surgical wound, interrupted sutures were placed using 4-0 silk thread to facilitate closure and healing (Figure 2E). The patient experienced no discomfort or pain during the procedure. The excised specimen (Figure 3) was placed in a formalin-filled tube and sent for histopathological examination, which confirmed the diagnosis of fibrolipoma, characterized by thick fibrous bands coursing through lobules of mature fat.

Postoperative care

The patient was provided with detailed post-surgical instructions. He was advised to avoid rinsing or gargling for the first 24 hours and instructed to refrain from brushing or applying pressure near the operative site. Antibiotics were not prescribed. An anti-inflammatory medication (ibuprofen 400-600 mg every four to six hours as needed), was recommended, ensuring the total daily dosage did not exceed 1200 mg. Additionally, a 0.12% chlorhexidine mouthwash was prescribed for use twice daily for one to two weeks to promote optimal healing and prevent infection.

Follow-up and healing

**Figure 4:**
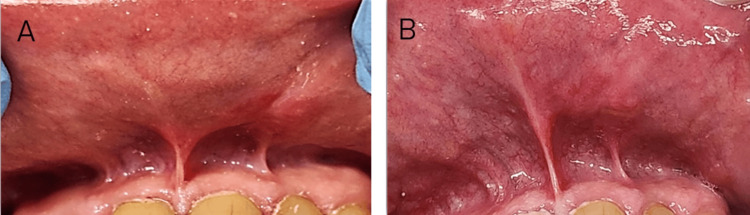
Postoperative healing of the lesion with no recurrence observed. A) Postoperative healing one month after surgery. B) Postoperative image of the lesion six months following surgery, showing complete healing and no signs of recurrence.

Follow-up evaluations were conducted after 24 hours, two weeks, one month, and six months post-surgery. Suture removal was performed at two weeks. The patient reported no pain or discomfort at the surgical site after two to four weeks. Complete healing of the oral lesion was observed without any complications, such as pain, bleeding, scarring, swelling, infection, or recurrence (Figure 4A). At the six-month follow-up, no recurrence was noted, and the patient expressed satisfaction with the treatment and outcomes (Figure 4B).

Clinical and patient-assessed outcomes

The treatment achieved excellent results, with complete restoration of the tissue and the anatomical architecture of the upper lip (restitutio ad integrum). The patient expressed high appreciation for the outcome and reported significant improvement in both aesthetics and functionality.

## Discussion

Intraoral lipomas of the lip are relatively rare but easily diagnosed due to their characteristic yellowish appearance and location near the mucosa. The differential diagnosis for these lesions includes fibroma, mucocele, salivary gland pathosis, and hemangiomas [9]. Typically, only a single lesion is observed [9, 10], though some cases report multiple lipomas, often associated with syndromes such as familial lipomatosis, Garner’s syndrome, and neurofibromatosis [10]. The etiology of lipomas could be explained by two theories: the hypertrophy theory, which suggests adipose tissue growth causes lipomas, and the metaplasia theory, which posits that aberrant mesenchymal cell distribution leads to lipomatous development [9, 10].

Surgical removal remains the treatment of choice for lipomas. Conventional excision methods, including electrosurgery, scalpel use, and cryosurgery, are associated with limitations such as delayed healing, excessive bleeding, pain, swelling, and discomfort [11]. However, the advent of lasers, particularly diode lasers, has significantly improved the excision of soft tissue lesions, especially in oral surgery. Diode lasers, first introduced in dentistry in 1999, are compact, portable, and relatively low-cost, making them highly accessible for dentists and oral surgeons [12]. These lasers offer several distinct advantages over traditional methods, including reduced tissue trauma, faster recovery, and enhanced precision, resulting in superior surgical outcomes [13, 14].

The present case report demonstrated that lipoma excision using a diode laser significantly increased effectiveness and reduced surgical time and postoperative complications. The postoperative period was uneventful, with healing occurring within a month. In a case series by Pai et al., various lipomas in the buccal cavity and gingiva were excised using diode lasers, with uneventful healing reported [2]. Similarly, the present case showed clear-cut excision, minimal bleeding, and excellent healing. However, due to the size of the lesion, sutures were required for closure.

Numerous studies have demonstrated the versatility and effectiveness of diode lasers in excising a wide range of oral soft-tissue lesions, including lipomas, fibrolipomas, pyogenic granulomas, irritational fibromas, squamous papillomas, and mucoceles. In the current case, the lipoma was successfully excised using a combination of diode laser and blunt scalpel techniques. Bakhtiari et al. also demonstrated the effective use of diode lasers for excising fibrolipomas from the mandibular retromolar pad area, utilizing laser and scalpel methods. This highlights the utility of diode lasers in removing lesions from challenging areas of the oral cavity [15]. Capodiferro et al. were the first to report the removal of fibrolipomas with diode lasers, suggesting them as the best alternative to conventional methods [16].

Diode lasers have proven to be particularly advantageous in challenging clinical scenarios, such as difficult-to-access areas, large lesions, and cases where significant bleeding is anticipated [5, 7, 12]. Their ability to provide precise tissue cutting, immediate hemostasis, and reduced bacterial contamination makes them an invaluable tool in these situations. Moreover, Pisano et al. demonstrated the successful removal of a pyogenic granuloma on the lower lip of an 11-year-old patient using a 980-nm diode laser, with no stress for the pediatric patient [17].

Furthermore, other oral soft-tissue lesions such as irritational fibromas, squamous papillomas, and mucoceles have also been excised using diode lasers, showing satisfactory healing within two to three weeks and minimal postoperative pain [18]. A 980nm diode laser has proven to be effective not only for soft-tissue oral lesions but also for the removal of large lipomas on the back, showcasing its versatility in treating various lipoma locations [19]. This also highlights their potential for application in a broader range of surgical procedures and specialties.

However, it is important to acknowledge the limitations of diode lasers, such as high costs and technique sensitivity. Improper use of diode lasers may lead to tissue trauma, emphasizing the need for adequate training and skill development among professionals utilizing this technology. When operated by skilled and experienced clinicians, diode lasers offer a minimally invasive and highly effective approach to the management of various oral soft-tissue lesions.

## Conclusions

This case demonstrates the effective removal of a 1.9 x 1.3 cm upper lip fibrolipoma using a 980nm diode laser with minimal bleeding and uneventful healing. It also showed a complete resolution within two to four weeks using only anti-inflammatory medication and full restoration of the lip architecture with no recurrence at the six-month follow-up. While surgical excision remains the traditional approach, our experience demonstrates that the 980nm diode laser offers a minimally invasive alternative for oral fibrolipomas, particularly beneficial in the labial region where tissue preservation and aesthetic outcomes are crucial. The successful outcome supports laser-assisted removal of oral soft tissue lesions as a viable treatment option, though standardized protocols considering lesion characteristics and anatomical location should be established through further clinical studies. Future studies are needed to assess long-term results and refine laser-assisted surgical procedures for standardized and evidence-based surgical techniques. 
